# Relationship between evenness and body size in species rich assemblages

**DOI:** 10.1098/rsbl.2013.0856

**Published:** 2013-12-23

**Authors:** A. E. Magurran, H. L. Queiroz, A. P. Hercos

**Affiliations:** 1 School of Biology, University of St Andrews, St Andrews, UK; 2 Mamirauá Institute for Sustainable Development, IDSM/MCTI, Tefe, Brazil

**Keywords:** diversity, relative abundance, várzea, freshwater fish, community structure

## Abstract

Evenness is a key measure of community structure. Here, we examine the relationship between evenness and size–abundance distributions for both individuals and species using data gathered from Amazonian fish assemblages. We show that evenness increases as the fraction of numerically abundant species in larger body-size classes rises. As any processes that enable larger bodied species to increase their numerical dominance will influence evenness, these results help explain why evenness is an important correlate of ecosystem function.

## 1. Introduction

The observation that all ecological assemblages include both common and rare species is so universal that it has become a law of ecology [1]. This variation in abundance, described as evenness, has been noted by many investigators including Darwin [2]. It is clear that some assemblages are very uneven, others less so, and that perfect evenness does not exist in nature. Cross-community comparisons reveal a complex relationship between evenness and species richness [3,4]. Evenness tends to decline as richness increases [5], but this pattern varies across assemblages. Research on plant communities [6,7–9] led Wilsey & Stirling [10] to conclude that richness and evenness are influenced by different processes.

**Figure 1.**
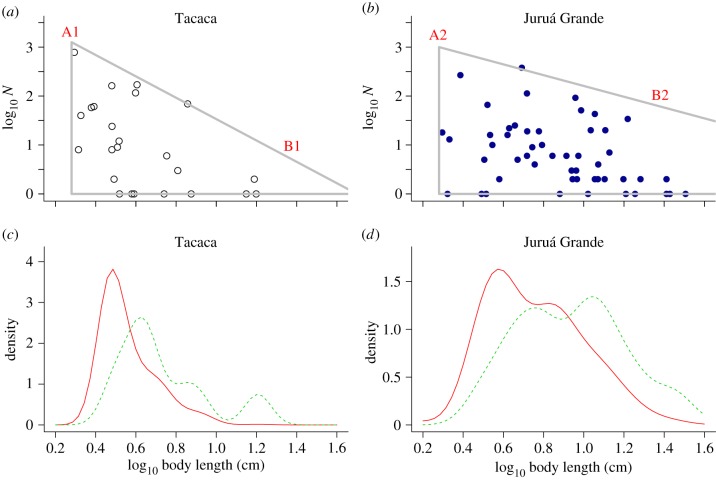
(*a*,*b*) The relationship between log numerical abundance and log body size in Tacaca pool and Juruá Grande lake, respectively. Dots depict species. As [11] notes, when plotted in this way, species in local communities occupy a roughly triangular space. As line A–B, which tracks the upper bound of this relationship, becomes shallower (A1/B1 versus A2/B2), the evenness of the assemblage will increase. A–B is an illustrative line, rather than a fitted one. Our goal here is not to identify a precise triangular relationship, but rather to show how the numerical abundance of larger species influences evenness. (*c*,*d*) Kernel density plots for two communities. Individual density plots are represented by the solid (red) line, and species density plots by the dashed (green) line.

One universal feature of assemblages is that they are uneven; another is that they are composed of species of different size. When log (numerical) abundance (or density) is plotted against log body size, the typical pattern (in local assemblages) is one where the points fall within a roughly triangular space [11,12]. The upper bound (see the illustrative line, A–B, in figure 1*a*,*b*) decreases from its maximum, typically monotonically, as body size increases. In local assemblages, the abundances of species of different body size will be influenced by a range of factors including predation and habitat complexity [13]. Any community process that enables more numerically abundant taxa to persist (regardless of their size class) will increase evenness. Using data from a diverse Amazonian fish assemblage [14], we test the specific hypothesis that a biologically plausible mechanism for this is when large-bodied species become numerically abundant.

## 2. Material and methods

### (a) Site description

This study was carried out at the Mamirauá Sustainable Development Reserve in Brazil's Central Amazon floodplain. The mosaic of forests, water bodies, lakes and channels [15] at Mamirauá is typical várzea (flooded forest) habitat and supports a diverse fish fauna [14,16]. The reserve's 1 124 000 ha are completely flooded for three to six months every year during the flooding pulse [14]. The *seca* or low water season occurs in the months of September, October and November. At this time, isolated lakes and forest pools appear.

Lakes contain floating meadow vegetation, a dense and structurally complex matrix of grass and macrophytes providing habitat cover for fish [17]. Forest pools lack floating meadow vegetation but can contain fallen branches and leaf litter. Although we did not measure habitat structure, previous work [18] suggests that the lake habitat will tend to be more structurally complex.

### (b) Sampling

We sampled 5 lakes (Araçázinho, Juruá Grande, Juruázinho, Tracajá and Pagão) monthly in the dry season (September–November) in 2003 and 2004; the methodology is described in detail elsewhere [14]. In brief, 16 m^2^ of vegetation was separated from the larger blocks of floating meadows, surrounded by a seine net (multi-filament; 2 mm mesh size, 30 m long and 6 m wide), and then lifted into a boat where the fish were collected. Total effort, which was consistent throughout, was 80 m^2^ of floating meadow per month per lake. Each of the six pools (Caxinguba, Jacareuba, Seringa, Tacaca, Taxizal and Urucurana) was sampled once during the dry season (2004 to 2006). All fish present in the pools were removed using three different methods: a cylindrical trap (*matapi* or *covo*), a seine net (as above) and a hand net.

All fish were transported to the field laboratory where they were identified [14] and measured. Whenever possible, fish were returned alive to the local water bodies. Species abundance and size data are provided in the electronic supplementary material.

### (c) Analysis

Olszewski [19] pointed out that the slope of the rarefaction curve [20] at its origin (its steepest point) is the same as Hurlbert's [21] probability of interspecific encounter (PIE). In essence, these approaches represent the probability that the next individual picked at random will belong to a different species. As such they provide a measure of evenness [22].

We produced individual rarefaction curves and calculated PIE [22] for each of our 11 assemblages. To show how individuals and species are distributed in relation to body length, we generated kernel density plots with the *sm* package [23] in R. We calculate MAD (the median absolute deviation from the median), using the R package *psych* [24], as a measure of statistical dispersion for these distributions (MADinds for individuals and MADspp for species). There is a shallower upper bound leading to more numerically abundant large-bodied species (upper right region of figure 1*b* versus 1*a*) when the distribution of body sizes is more dispersed and less peaked—MAD is a robust measure of this dispersion and resilient to outliers [25].

## 3. Results

Rarefaction curves (see the electronic supplementary material) show that the assemblages vary in their evenness (as reflected in the steepness of the initial portion of the curve). The kernel density plots (figure 1*c*,*d*; electronic supplementary material) reveal that individuals are concentrated in a narrower range of body sizes than species, and that this is particularly true for the pools, although Urucurana is an exception. Thus, while the species in each of these assemblages are distributed across a range of body sizes, individuals are typically more similar in size and usually small. There is however heterogeneity in the individual density plots, and this is linked to evenness.

**Figure 2.**
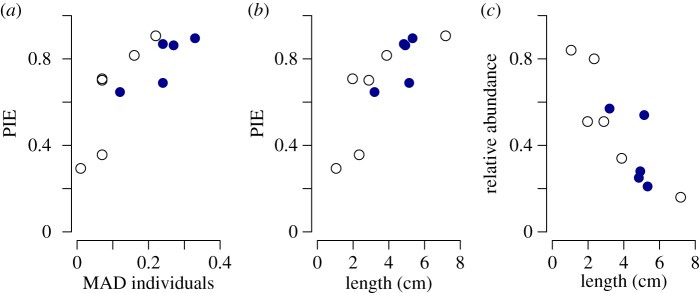
Relationship between (*a*) PIE and MAD (for individuals), (*b*) PIE and the length of the dominant species in centimetre and (*c*) the relative abundance of the dominant species and the length of the dominant species in centimetre. Pools are shown as black (unfilled) circles and lakes as blue (open) circles.

PIE is strongly correlated with the dispersion of individuals among size classes (MADinds) (*r* = 0.79, *p* = 0.0031; figure 2*a*) but not with the dispersion of species among the same size classes (MADspp) (*r* = 0.03, *p* = 0.92). The dispersion of individuals (MADinds) is strongly correlated with the size of the dominant species (*r* = 0.86, *p* = 0.006; figure 2*b*), which is in turn negatively correlated with the relative abundance of the dominant species (*r* = −0.84, *p* = 0.001; figure 2*c*). All significant correlations remain significant (*p* < 0.05) if a Bonferroni correction is applied. (Other measures of evenness, and using the interquartile range as a measure of dispersion in place of MAD, produce the same result: see the electronic supplementary material for details.)

## 4. Discussion

These analyses reveal that there is variation in evenness in Amazonian fish assemblages and that this variation can be linked to the distribution of individuals among size classes. In essence, the shallower the decline in maximum species abundance across size classes, the more even the assemblage. This is reflected in the fact that in these more even assemblages dominant species tend to be larger, as well as representing a smaller fraction of total dominance. Although this result may seem obvious in retrospect, it provides a novel link between evenness and body-size distributions.

Any processes that modulate the slope of the upper bound relationship between maximum abundance and size class are thus expected to influence assemblage evenness. Habitat complexity is one factor that is likely to enable increased abundances across a range of size classes. Cotgreave & Harvey [26] showed in a comparative analysis that assemblages with greater habitat complexity tend to be more even. While we did not explicitly measure habitat complexity, the floating meadow habitats in lakes would be expected to be more structurally diverse than the forest pools. On the other hand, evenness would be reduced in cases where larger bodied taxa are disadvantaged, for example in polluted or disturbed localities. Indeed, the ABC (abundance/biomass comparison) method [27] of community assessment argues that stressed communities can be identified by a shift in the rank abundance plots of numerical abundance relative to biomass brought about by a reduction in the relative abundance of the larger bodied taxa. The different sampling protocols used here could in part explain the differences between lakes and pools, but we stress that our goal is not a comparison of these habitats *per se* but rather we make use of their characteristics to illustrate how the distribution of size classes in an assemblage influences evenness.

Evenness statistics quantify the relative abundance of species. Evenness will thus increase if there are many equally abundant species no matter the size class. However, factors, such as competition and the fractal structure of the environment [13], make it unlikely that many common species (i.e. the species that increase the evenness of a community) will belong to any single size class. It is for this reason that evenness is linked to the body-size effects apparent in the individual density plots, rather than the species density ones.

It is increasingly clear that ecosystem function is underpinned by evenness as well as species richness [28–30]. The link with the distribution of body size identified here sheds light on the processes that drive this relationship. Our analysis shows the insights that can be gained from examining species abundance distributions from the perspective of labelled species [1], and allows predictions about how assemblage evenness will change as a result of manipulations, and how these changes will in turn mediate function.
